# Real-World Lab Data in Natalizumab Treated Multiple Sclerosis Patients Up to 6 Years Long-Term Follow Up

**DOI:** 10.3389/fneur.2018.01071

**Published:** 2018-12-07

**Authors:** Maxi Kaufmann, Rocco Haase, Undine Proschmann, Tjalf Ziemssen, Katja Akgün

**Affiliations:** MS Center Dresden, Center of Clinical Neuroscience, Carl Gustav Carus University Hospital, University of Technology Dresden, Dresden, Germany

**Keywords:** natalizumab, multiple sclerosis, real-world lab data, peripheral immune cell subtypes, clinical practice

## Abstract

Natalizumab inhibits the transmigration of immune cells across the blood-brain barrier thus inhibiting inflammation in the central nervous system. Generally, this blockade at the blood-brain barrier has significant influence on the circulating lymphocytes. Up to date, only short-term data on peripheral blood parameters are available which are mostly from controlled clinical trials and not from real-world experience. Real-world lab data of 120 patients diagnosed with highly active disease course of relapsing-remitting multiple sclerosis (RRMS) were analyzed during natalizumab treatment. Patient sampling was performed by consecutive recruitment in the Multiple Sclerosis Center Dresden. Lab testing was performed before and at every third infusion up to 72 months follow-up. After first natalizumab infusion, absolute numbers of all major lymphocyte populations including CD4+ T-cells, CD8+ T-cells, CD19+ B-cells, and NK-cells significantly increased and remained stable during the whole observation period of 72 months. Upon lymphocyte subsets, CD19+ B-cells presented a disproportionate increase up to levels higher than normal level in most of the treated patients. Neutralizing antibodies to natalizumab abrogated the described changes. Intra-individual variation of lymphocytes and its subsets remained in a narrow range for the whole treatment period. CD4/CD8 ratio did not change compared to baseline measurement up to 6 years of natalizumab treatment. Monocytes, eosinophils, and basophils, but not neutrophils persistently increased during natalizumab treatment. Hematological parameters including erythrocyte, platelet count, hemoglobin, and hematocrit remained unchanged compared to baseline. Interestingly, immature precursor cells including erythroblasts were detectable in 36,8% of the treated patients during natalizumab therapy, but not in the pretreatment period. Asymptomatic elevations of liver enzymes were rare, mostly only transient and lower than 3x upper normal limit. Kidney function parameters remained stable within physiological ranges in most patients. CRP levels >20 mg/dl were recognized only in 10 patients during natalizumab therapy and were mostly linked to respiratory tract infections. In our present analysis, we report persistent, but stable increases of peripheral immune cell subtypes in natalizumab treated patients. Additional serological analyses confirm excellent tolerability and safety even 6 years after natalizumab initiation in post-marketing experience.

## Introduction

Natalizumab (NAT) is a humanized monoclonal antibody selectively directed against the α4-subunit of the very late antigen-4 (VLA-4) integrin, a specific adhesion molecule on the surface of leukocytes except neutrophils. The α4-integrin interacts with the vascular cell adhesion molecule-1 (VCAM-1) expressed on endothelial cells of blood vessels to mediate extravasation and transmigration of immune cells across the blood-brain barrier into the central nervous system (CNS) (1–3). By its unique mechanism of action, NAT blocks the VLA-4/VCAM-1 mediated leukocyte-endothelial interaction (2, 4). Though, lymphocyte migration into brain tissue is prevented and the CNS inflammation is inhibited (5). NAT is highly effective in relapsing-remitting (RR) multiple sclerosis (MS) proven by marked decrease in relapse rate, MRI activity and disease progression (6, 7). There is a growing experience in selection of appropriated patients and use of NAT in everyday clinical practice since approval. Among disease-modifying treatments (DMT) in MS therapy, NAT is associated with an increased risk of progressive multifocal leukoencephalopathy (PML) especially in John Cunningham virus (JCV) positive and long-term treated patients (8). Since re-approval in 2006, a detailed and standardized management program was established which is now used in everyday clinical practice (8–10). As part of this risk management plan, standardized lab testing is recommended including complete blood count, peripheral immune cell status, and serological parameters to be aware of clinical relevant changes. Especially lymphocytosis has been already described in NAT-treated patients due to impaired lymphocyte extravasation into tissues as well as mobilization of hematopoetic precursor cells from bone marrow (11–16). As part of our NAT management, data from clinical practice are collected providing longitudinal information on different outcomes inclusive adverse affects (17). Such real-world evidence (RWE) and observational studies are becoming increasingly popular because they reflect the usefulness of drugs in real life and have the ability to discover uncommon or rare adverse drug reactions inclusive lab abnormalities (18). Therefore, RWE can assist to evaluate the drug profile in clinical practice and to link it with other clinical outcomes. The MSDS3D software which has been adapted to the NAT management in particular combines documentation of patient data with management of patients with MS implementing treatment-specific modules, to collect data of safety management inclusive lab data with regard to the characteristics of different treatments and populations (19).

Today, NAT has been successfully used in MS patients for more than 11 years. The increase in lymphocyte count was already discussed during the initial clinical trials (6, 20). Further single evaluations followed after approval and in post-marketing experience (21–24). Nevertheless, a systematic and long-term evaluation of real-world routine lab testing data including peripheral cell subtypes and serological parameters is not yet available.

In this study, we present real world laboratory data of a cohort of NAT treated patients up to 72 months follow up. We aim to describe the biological impact of NAT on peripheral blood cell subset distribution during long period treatment observation in the real world. Additional serological analyses allow evaluation of tolerability and safety after NAT initiation in real world experience.

## Methods

### Patients

**Figure 1:**
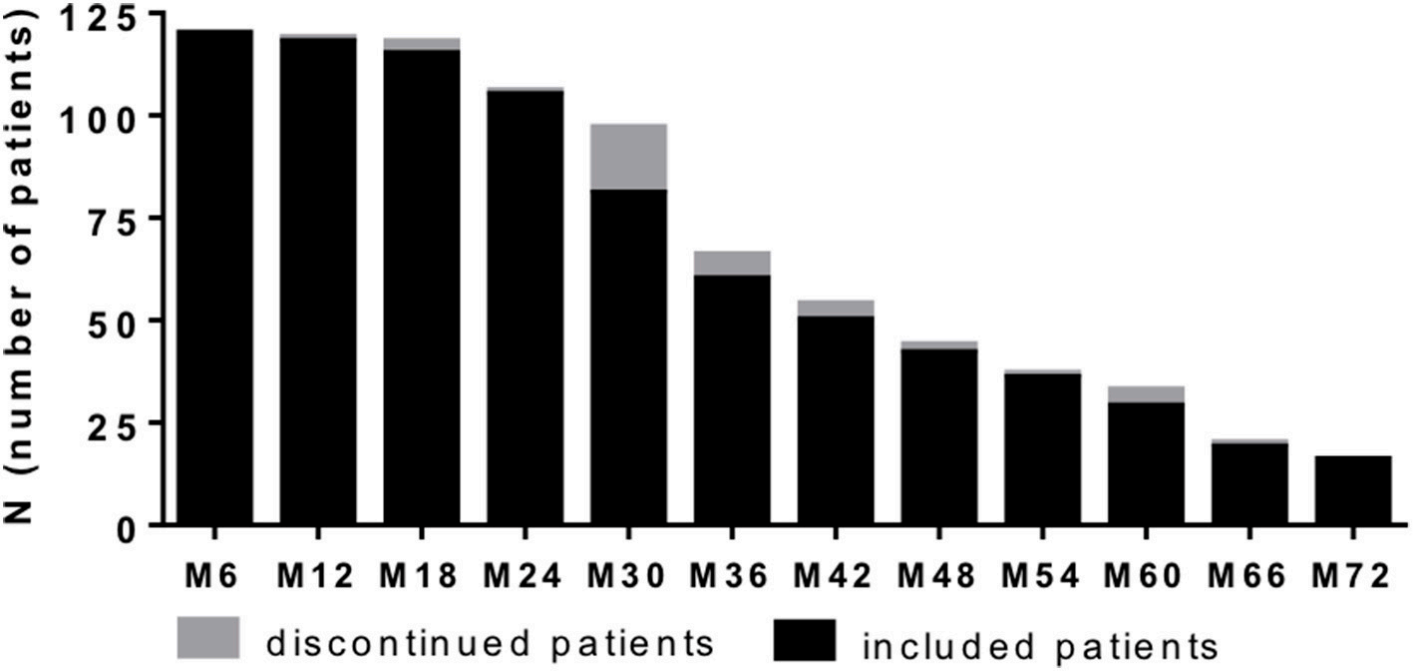
Patient recruitment per 6 months period. One hundred and twenty patients with highly active MS were recruited and evaluated at data analysis. Based on consecutive sampling some of patients already reached 72 month follow up, whereas others did not yet at time point of data analysis. Number of patients included at defined time point are depicted (black bar). Additionally number of patients that discontinued NAT treatment at defined time point are presented (gray bar). Clinical and lab data were evaluated every 3 months.

**Figure 2:**
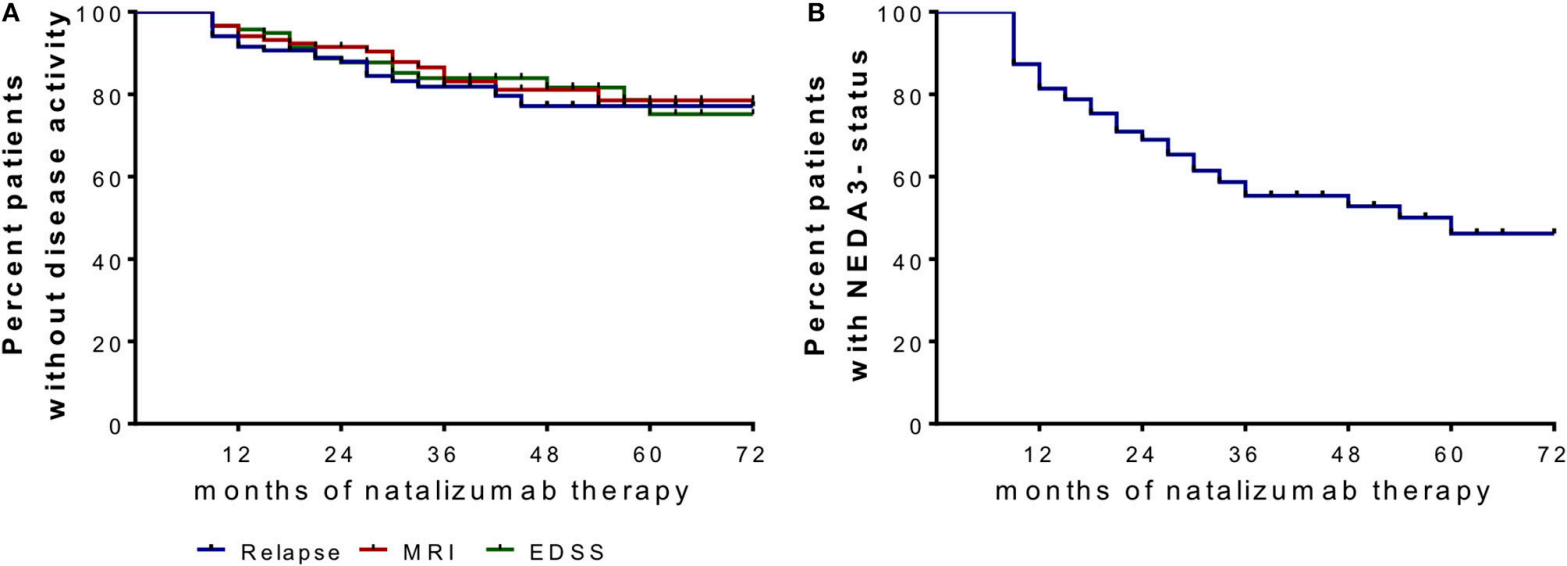
Proportion of patients without disease activity and NEDA-3 status after NAT start. **(A)** Clinical parameters are depicted in a Kaplan–Meier survival curve analysis for relapses, confirmed EDSS progression (≥1.0 point increase if EDSS baseline score was < 4.0; ≥0.5 point increase if EDSS baseline score was ≥4.0) and MRI progression (one or more new T2 or gadolinium enhancing lesions) after month 6 and during 72 months follow up. **(B)** No evidence of disease activity (NEDA)-3 status was confirmed when criteria of no relapses, no EDSS progression and no MRI progression were met.

We included 120 patients (91 females and 29 males) diagnosed with RRMS according the McDonald criteria and highly active disease course (25). After critical review of clinical and MRI data as well as available treatment options, NAT treatment was initiated. Patient sampling was performed by consecutive recruitment (Figure 1) in the MS center, University Hospital Dresden. Expanded Disability Status Scale (EDSS) was performed by an experienced, Neurostatus-certified neurologist (26). Patient baseline characteristics are reported in Table 1. Patients starting with NAT presented at an mean age about 33.7 ± 9.6 years, an median EDSS score about 3.5 and median duration since diagnosis of RRMS about 4.0. About 69.2% of patients received at least one DMT before NAT initiation, 30.8% patients were initiated to NAT without previous DMT. During a standardized treatment switch procedure, injectables were stopped 2 weeks before natalizumab start, fingolimod was stopped 8 weeks before natalizumab and other DMTs at least 6 months before natalizumab initiation. The proportion of patients without disease activity and NEDA-3 status defined by no relapses, no confirmed EDSS progression (≥1.0 point increase if EDSS baseline score was < 4.0; ≥0.5 point increase if EDSS baseline score was ≥4.0), and no MRI progression (one or more new T2 or gadolinium enhancing lesions) during the 72 months observation period are presented in Figure 2. Data have been collected from the MSDS3D database. The study was approved by the institutional review board of the University Hospital of Dresden. Patients gave their written informed consent.

**Table 1:** Baseline characteristics.

**Age (yr ± SD)**	**33.7 (9.6)**
Male-[no. (%)]	29 (24.2)
Female-[no. (%)]	91 (75.8)
Previous use DMT-[no. (%)]	83 (69.2)
Interferon-beta	51 (42.5)
Glatiramer acetate	21 (17.5)
Fingolimod	4 (3.3)
Other approved medications	7 (5.8)
none-[no. (%)]	37 (30.8)
Duration since MS diagnosis (mean yr ± SD)	5.6 (5.8)
Mean score on EDSS	3.8 (1.8)

*Baseline characteristics of evaluated patients. Other approved medications include, Triamcinolon (1), Azathioprin (1), Mitoxantron (4), Laquinimod (1). DMT, disease modifying therapy; yr, years; SD, standard deviation; no, number*.

### NAT Infusion Protocol and Blood Sampling

NAT infusion protocol used in our MS center was adapted to the standardized infusion protocol described in guidelines of diagnosis and treatment of MS patients of the German association of MS (27). NAT (300 mg) was given every 4 weeks intravenously (i.v.) over the course of 1 h. According to our standard operation procedure, routine blood analysis and EDSS evaluations were realized before NAT start and every 3 months. In this analysis, we evaluated these parameters up to 72 months follow up.

### Routine Blood Analysis

Standardized blood testing was performed for routine blood parameters at the Institute of Clinical Chemistry and Laboratory Medicine, University Hospital in Dresden, Germany. The institute complies with standards required by DIN-EN-ISO-15189:2014 for medical laboratories. Routine blood testing included complete blood cell count, liver enzymes, creatinine, sodium, potassium, and C-reactive protein (CRP). Serological testing for JCV antibodies was performed before natalizumab start and every 6 months follow up and was measured at Unilabs, Denmark (Stratify JCV^TM^ Test).

### Immune Cell Phenotyping by Fluorescence-Activated Cell Sorting (FACS)

After blood collection, subpopulations of T-cells, B-cells, and natural killer (NK) cells were characterized by surface staining with fluorescence labeled anti-CD3, anti-CD4, anti-CD8, anti-CD16, anti-CD14, anti-CD19, anti-CD56 (BD Biosciences, Heidelberg, Germany) according to the manufacturer's instructions. Negative controls included directly labeled or unlabeled isotype-matched irrelevant antibodies (BD Biosciences). Cells were evaluated on FACSCanto II flow cytometer.

### Statistical Analysis

**Figure 3:**
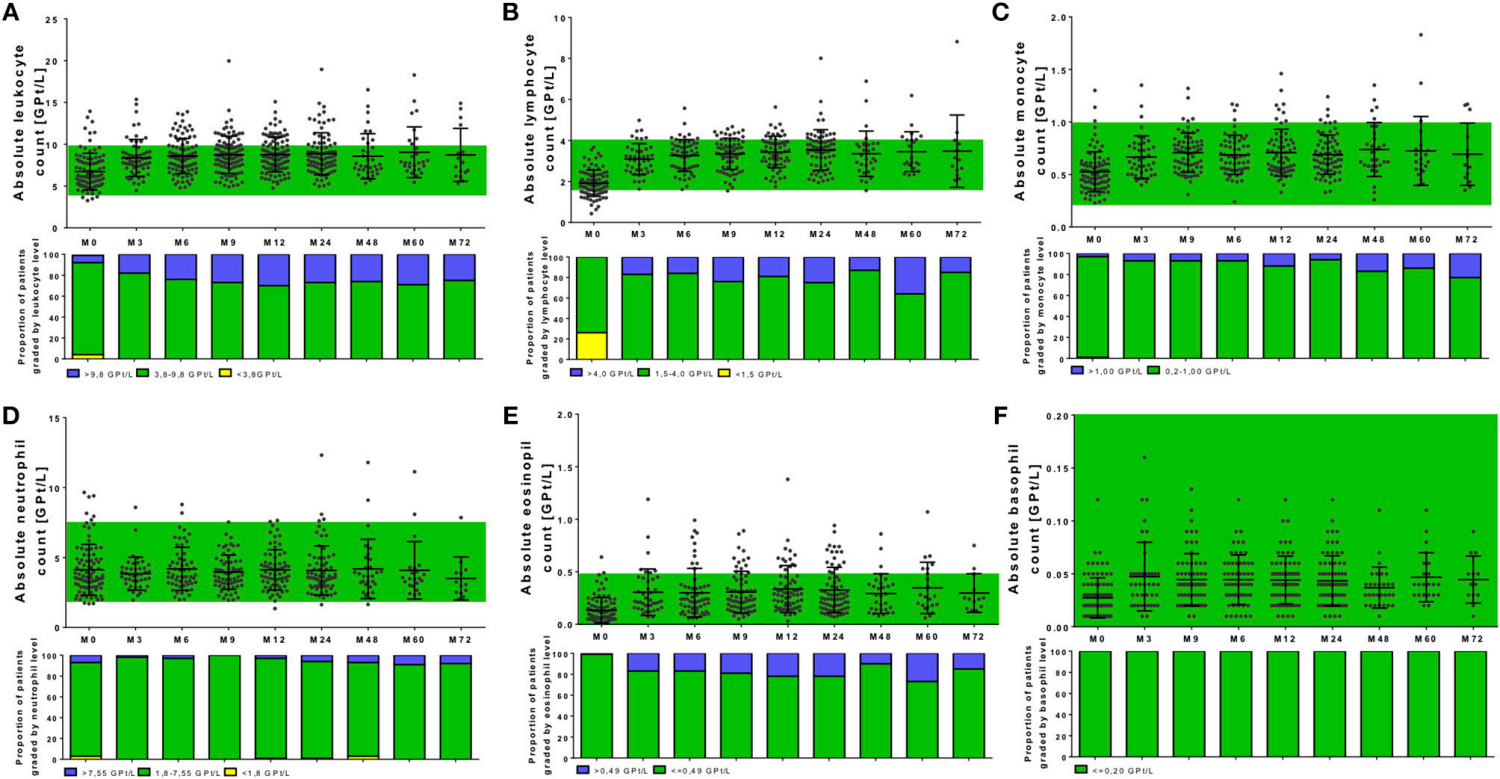
Complete blood analysis during NAT treatment. Blood samples were taken before NAT started (M0) and every 3 months. Blood samples were analyzed in a certified medical laboratory. Analysis of complete blood count as leukocytes **(A)**, lymphocytes **(B)**, monocytes **(C)**, neutrophils **(D)**, eosinophils **(E)**, and basophils **(F)** were included. Data are shown for baseline analysis (M0), month 3 (M3), month 6 (M6), month 12 (M12), and every 12 months follow up (M24, M48, M60, M72). Results are depicted as mean ± standard deviation. Green background indicates reference range of depicted parameters. Additionally, proportion of patients graded by white blood cell levels are shown: lower than reference range (yellow), reference range (green), and higher than the reference range (blue). Levels of significance are presented in Table 2.

**Figure 4:**
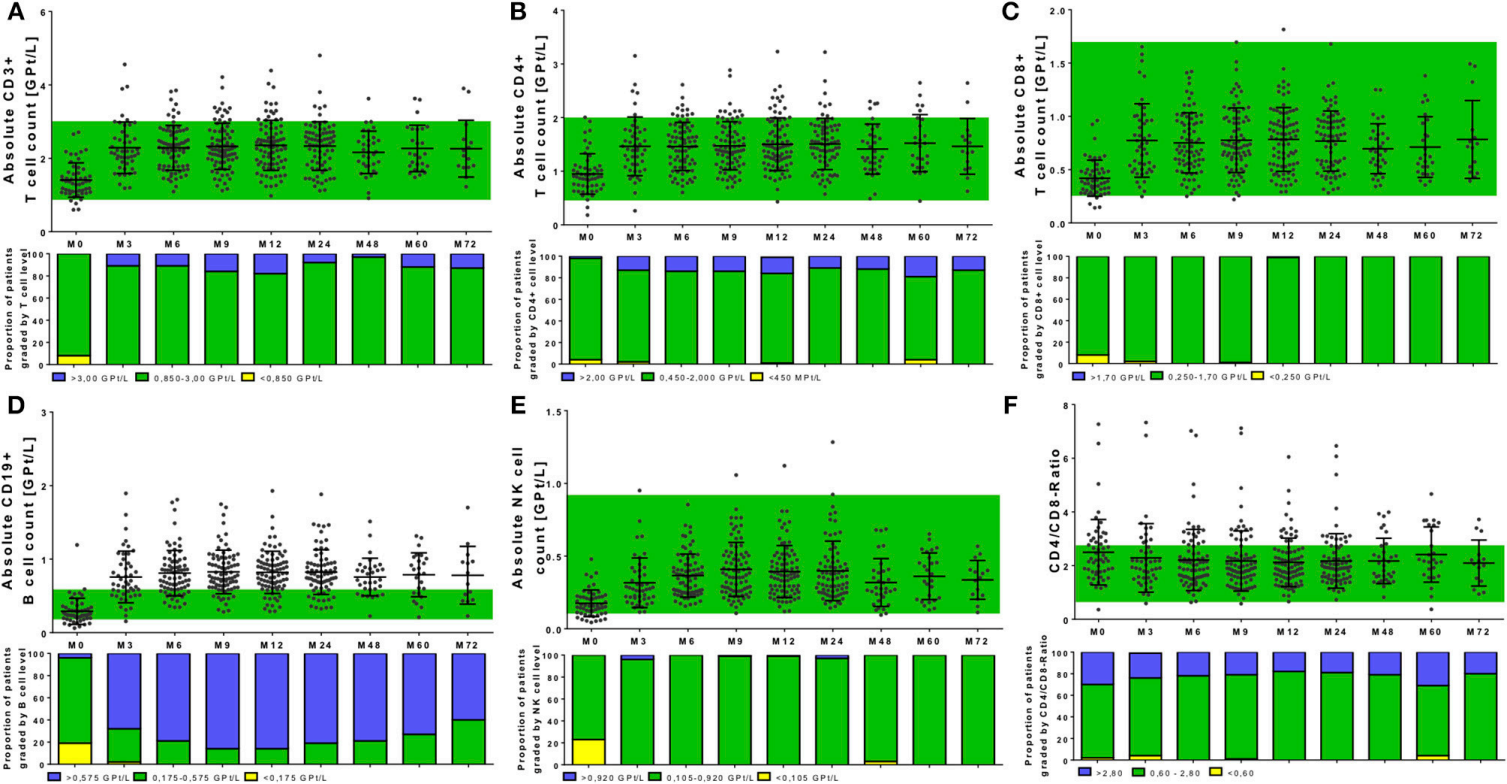
Cell count of selective immune cell subpopulations during NAT treatment. Absolute cell count of CD3^+^ T cells **(A)**, CD4^+^ T cells **(B)**, CD8^+^ T cells **(C)**, CD19^+^ B cells **(D)**, NK cells **(E)**, and the CD4^+^/CD8^+^-Ratio **(F)** are depicted. Data are shown for baseline analysis (M0), month 3 (M3), month 6 (M6), month 12 (M12), and every 12 months follow up (M24, M48, M60, M72). Mean ± standard deviation are presented. Green background indicates reference range of depicted parameters. Additionally, proportion of patients graded by lymphocyte subtype level are shown: lower than reference range (yellow), reference range (green) and higher than the reference range (blue). Level of significance are presented in Table 2.

**Figure 5:**
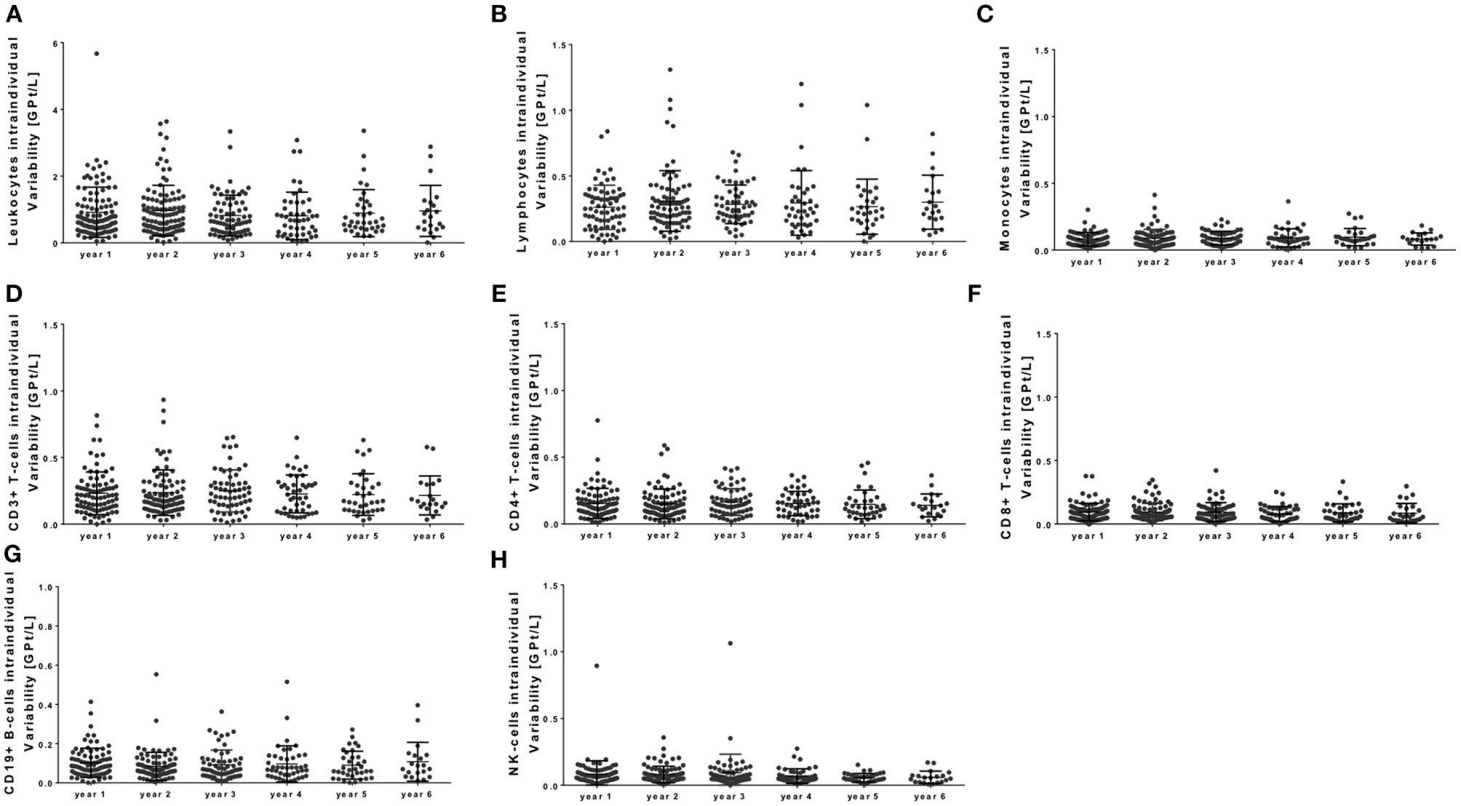
Intra-individual variability of peripheral immune cell subsets during NAT treatment. Intra-individual variation of leukocytes **(A)**, lymphocytes **(B)**, monocytes **(C)**, CD3+ T cells **(D)**, CD4+ T cells **(E)**, CD8+ T cells **(F)**, CD19+ B cells **(G)**, and NK cells **(H)** are depicted for each year. Intra-individual variability was defined as the standard deviation of absolute cell counts every 3 months in every year after NAT start (starting at month 3). Mean ± standard deviation.

Data were analyzed applying Generalized Linear Mixed Models with Gamma distribution and log link function due to the right-skewed distribution pattern of the data. Bonferroni correction for pairwise tests was used. Correlations were calculated using Spearman's correlation model. At the time, there were no clear variables at hand that should have been treated as confounders. Since we had no determining groups to test for differences, confounders over time were left to be considered. In the graphs (Figures 3–**5**, **7**), data are given as mean ± standard deviation (SD). In Table 2, data are additionally presented as mean ±95% confidence interval (CI) providing information about the precision of estimates over the course of the study. Values of ^*^p < 0.05, ^**^p < 0.01, and ^***^p < 0.001 were considered significant.

**Table 2:** Levels of statistical significance.

**Time point**	**Leukocyte**	**Lymphocyte**	**Monocyte**	**Neutrophile granulocyte**	**Eosinophile granulocyte**	**Basophile granulocyte**	**CD3+ Tcell**	**CD4+ Tcell**	**CD8+ Tcell**	**CD19+ Been**	**NK cell**
MO	6.73 (6.30; 7.18)	1.91 (1.78; 2.05)	0.53 (0.49; 0.57)	4.13 (3.77; 4.52)	0.13 (0.11; 0.16)	0.03 (0.02; 0.03)	1.41 (1.29; 1.54)	0.95 (0.85; 1.06)	0.42 (0.38; 0.47)	0.29 (0.25; 0.34)	0.18 (0.15; 0.20)
M3	8.37 (7.83; 8.95), *p* < 0.001	3.09 (2.87; 3.33), *P <* 0.001	0.67 (0.61; 0.73), *p* = 0.005	3.85 (3.51; 4.22), *P =* 1.000	0.34 (0.26; 0.45), *p* = 0.001	0.05 (0.04; 0.06), *P =* 0.007	2.29 (2.10; 2.50), *p* < 0.001	1.46 (1.32; 1.63), *p <* 0.001	0.77 (0.68; 0.88), *P <* 0.001	0.76 (0.66; 0.86), *p <* 0.001	0.36 (0.27; 0.47), *P =* 0.011
M6	8.56 (8.18; 9.03), *p* < 0.001	3.26 (3.07; 3.47), *P <* 0.001	0.69 (0.64; 0.74), *p* < 0.001	4.19 (3.81; 4.60), *P =* 1.000	0.34 (0.26; 0.44), *p* = 0.001	0.04 (0.04; 0.05), *P <* 0.001	2.29 (2.15; 2.43), *p* < 0.001	1.46 (1.36; 1.57), *p <* 0.001	0.75 (0.69; 0.82), *P <* 0.001	0.81 (0.74; 0.88), *p <* 0.001	0.37 (0.33; 0.40), *P <* 0.001
M9	8.74 (8.33; 9.18), *p* < 0.001	3.35 (3.17; 3.54), *P <* 0.001	0.71 (0.67; 0.75), *p* < 0.001	3.95 (3.67; 4.26), *P =* 1.000	0.34 (0.27; 0.42), *p* < 0.001	0.04 (0.04; 0.05), *p <* 0;001	2.33 (2.19; 2.47), *p <* 0.001	1.47 (1.38; 1.57), *p <* 0.001	0.78 (0.71; 0.85), *P <* 0.001	0.83 (0.76; 0.89), *p <* 0.001	0.41 (0.37; 0.45), *P <* 0.001
M12	8.77 (8.37; 9.18), *p* < 0.001	3.43 (3.25; 3.62), *P <* 0.001	0.71 (0.66; 0.76), *p* < 0.001	4.12 (3.78; 4.48), *P =* 1.000	0.36 (0.30; 0.43), *p* < 0.001	0.04 (0.04; 0.04), *P <* 0.001	2.35 (2.21; 2.51), *p* < 0.001	1.50 (1.40; 1.61), *p <* 0.001	0.78 (0.72; 0.85), *P <* 0.001	0.84 (0.77; 0.92), *p <* 0.001	0.39 (0.36; 0.43), *P <* 0.001
M24	8.86 (8.35; 9.41), *p* < 0.001	3.54 (3.31; 3.77), *P <* 0.001	0.69 (0.65; 0.73), *p* < 0.001	4.08 (3.69; 4.50), *P =* 1.000	0.33 (0.28; 0.38), *p* < 0.001	0.04 (0.04; 0.05), *P <* 0.001	2.33 (2.19; 2.49), *p* < 0.001	1.51 (1.40; 1.62), *p <* 0.001	0.77 (0.71; 0.84), *P <* 0.001	0.82 (0.76; 0.90), *p <* 0.001	0.40 (0.36; 0.45), *P <* 0.001
M48	8.57 (7.72; 9.51), *p* = 0.010	3.35 (2.98; 3.77), *P <* 0.001	0.74 (0.65; 0.84), *p* = 0.001	4.19 (3.49; 5.02), *P =* 1.000	0.29 (0.23; 0.37), *p* = 0.001	0.04 (0.03; 0.04), *p =* 0.512	2.16 (1.97; 2.37), *p* < 0.001	1.41 (1.26; 1.58), *p <* 0.001	0.70 (0.62; 0.78), *P <* 0.001	0.83 (0.68; 1.02), *p <* 0.001	0.32 (0.27; 0.38), *P =* 0.001
M60	9.04 (7.98; 10.24), *p* = 0.006	3.45 (3.07; 3.88), *P <* 0.001	0.73 (0.60; 0.88), *p* = 0.193	4.09 (3.31; 5.04), *P =* 1.000	0.35 (0.26; 0.47), *p* = 0.002	0.05 (0.04; 0.06), *P =* 0.009	2.27 (2.04; 2.53), *p* < 0.001	1.52 (1.33; 1.74), *P <* 0.001	0.71 (0.61; 0.83), *P <* 0.001	0.92 (0.68; 1.25), *p <* 0.001	0.36 (0.30; 0.43), *P <* 0.001
M72	8.73 (7.30; 10.43), *p* = 0.448	3.48 (2.64; 4.58), *p =* 0.047	0.69 (0.55; 0.88), *p* = 1.000	3.50 (2.76; 4.45), *P =* 1.000	0.30 (0.21; 0.42), *p* = 0;051	0.04 (0.03; 0.06), *P =* 0.239	2.26 (1.90; 2.69), *p* = 0.002	1.46 (1.22; 1.75), *p =* 0.011	0.78 (0.62; 0.99), *P =* 0.006	1.04 (0.62; 1.77), *p =* 0.226	0.34 (0.28; 0.412), *P =* 0.001

*Mean and 95% confidence interval of each time-point of selected immune cell subsets. Level of significance with respective p-value (change compared to MO)*.

## Results

### Complete Blood Cell Count and Its Variation During Long-Term NAT Therapy

In our analysis 120 patients were included starting on NAT therapy. Before NAT initiation, a wide range of leukocyte resp. lymphocyte counts (3.3–13.9 GPT/L, respectively, 0.4–3.7 GPT/L) was seen (Figures 3A,B, Table 2). Levels of baseline leukocyte resp. lymphocyte count did not significantly differ between patients without or with different previous DMT use in our cohort. At NAT start, 24 patients presented with lymphocyte counts < 1.5 GPT/L without a specific pretreatment pattern.

After NAT initiation, leukocyte and lymphocyte counts increased significantly (global time effect *p* < 0.001, Figures 3A,B, Table 2). On average, leukocyte count increased up to about 2.05 GPT/L absolutely and 37.33% relatively, lymphocytes up to about 1.63 GPT/L in absolute and 99.95% in relatively. The highest increase in leukocyte and lymphocyte count was seen in patients pretreated with fingolimod (only 4 patients in our cohort included) before NAT start. Furthermore, there were no significant differences in leukocyte, respectively, lymphocyte increase or distribution during NAT treatment depending on pretreatment use. NAT treatment led to counts even higher than predefined upper normal limit for leukocytes in 28.8% of the treated patients and for lymphocytes in 21.4% of treated patients. Persistent lymphopenia < 1.5 GPT/L was not seen during NAT treatment in our cohort.

Other leukocyte subtypes including monocytes, eosinophils, and basophils were significantly elevated and again increased even higher than upper normal level (global time effect *p* < 0.001, Figures 3C,E,F, Table 2). These effects were persistent without significant fluctuations during the entire observation period in all treated patients. Interestingly, no changes were seen regarding absolute number and distribution of neutrophils during the whole observation period (global time effect *p* = 0.863, Figure 3D, Table 2). Additional evaluation of blood parameters including hemoglobin, hematocrit, erythrocyte count, and platelets demonstrated stable parameters in normal range during the 72 months evaluation period. Immature precursor cells including erythroblasts were detectable in 36.8% of the treated patients during NAT therapy, whereas none of these patients presented erythroblasts in the pretreatment period.

### Effects on Distinct Lymphocyte Subsets and Its Intra-Individual Variation

In addition to complete blood count analysis, distinct lymphocyte subsets were evaluated. Absolute count of T cell subtypes presented in the physiological reference range in most of the patients before NAT start (Figures 4A–C). Only in single patients, T cells below lower normal limit could be identified (two patients without pretreatment, one patient with previous fingolimod treatment, one patient with previous interferon-beta treatment). Interestingly, evaluating predefined reference ranges for B cells and NK cells, most of the patients with lower lymphocyte count at baseline were associated with lower B cell and NK cell count at baseline as well (Figures 4D,F). All investigated cell subtypes including CD3+ T cells, CD4+ T cells, CD8+ T cells, CD19+ B cells, and NK cells, significantly increased after NAT initiation (global time effect *p* < 0.001, Figures 4A–E, Table 2). A higher portion of patients with a more intense increase of CD8+ T cells was seen compared to CD4+ T cells (Figures 4A–C). For CD4+ T cells, an increase >10% was seen in 94.2% of treated patients, an increase >20% was seen in 88.5% of treated patients, an increase >50% was seen in 61.5% of treated patients, and an increase >100% was seen in 23.1% of treated patients. In contrast for CD8+ T cells, an increase >10% was found in 96.2%, an increase >20% was found on 92.3% of patients, an increase >50% was seen in 80.8% of patients, and an increase >100% was found in 38.5% of NAT treated patients. Nevertheless, CD4/CD8 ratio was not significantly changed in our cohort during long-term NAT treatment (Figure 4F). Patients with increased CD4/CD8 ratio at baseline presented increased CD4/CD8 ratio during follow up as well. CD19+ B cells presented the most pronounced increase in their frequency distribution in almost all treated patients (Figure 4D, Table 2). Regarding CD19+ B cells, all patients increased more than 20%. An increase >50% was seen in 94.2% of treated patients and an increase >100% was seen in 86.5 % of treated patients. Additionally, NK cells markedly increased, remained in the reference range with stable levels during the whole period of investigation (Figure 4E, Table 2). An increase >20% was seen in 98.1% of treated patients, an increase >50% was seen in 90.4% of treated patients and an increase >100% was seen in 63.5% of treated patients.

There was a strong correlation between baseline count and relative increase for lymphocytes and its subtypes [*r* – (0.792 – 0.625); *p* < 0.0001], but no significant correlation between baseline count and absolute increase. Age [*r* – (0.221 – 0.109); *p* > 0.05] and different pretreatment conditions [*r* –(0.039 – 0.052); *p* > 0.05] could not predict changes and pattern of immune cell subtypes during NAT therapy. In addition, intra-individual variability of different immune cell subtypes was evaluated during NAT treatment (Figure 5). Intra-individual variability was defined as the standard deviation of absolute cell count every 3 months in every year after NAT start. Most pronounced variability was seen for the leukocyte count, whereas lymphocytes and its subtypes varied only in a narrow range (Figure 5).

### Reference to Clinical Parameters

The proportion of patients with or without disease activity and NEDA-3 status are presented in Figure 2. Evaluation of immune cell profiles in NEDA-3 positive patients vs. active patients could not confirm significant differences in the immune cell profile of leukocytes, lymphocytes, monocytes, T cell subsets, B cells, or NK cells during NAT treatment.

At NAT start, JCV antibody status was negative in 75 patients, positive in 19 patients, and unknown in 26 patients. During NAT treatment, 25.4% of JCV antibody negative patients switched to JCV antibody positive status. There were no cases of PML in our cohort. During the observation period, 39 patients stopped NAT treatment: 35 patients stopped because of positive JCV antibody status, three patients because of efficacy reasons and one patient because of the individual patient wish. Immune cell profile in patients that terminated NAT treatment was not statistically different to patients continuing NAT therapy.

### Lymphocytes in a NAT Treated Patient With Neutralizing Antibodies Against NAT (nAbs)

**Figure 6:**
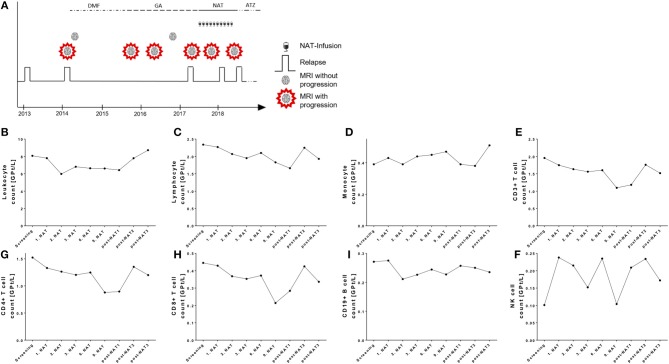
Case presentation and course of peripheral immune cell subset in a NAT treated patient with positive nAbs. **(A)** Clinical data including relapse activity, MRI progression, and treatment conditions are presented. DMF, dimethylfumarate; GA, glatiramer acetate; NAT, natalizumab; ATZ, alemtuzumab. **(B–I)** Absolute cell count of leukocytes **(B)**, lymphocytes **(C)**, monocytes **(D)**, CD3+ T cells **(E)**, CD4+ T cells **(F)**, CD8+ T cells **(G)**, CD19+ B cells **(H)**, and NK cells **(I)** are depicted. Data are shown for screening, NAT treatment period (1. NAT, 1st NAT infusion etc), and period after NAT cessation (post-NAT1, post-NAT2, post-NAT3; 1, 2, or 3 months after NAT stop).

This female patient was diagnosed RRMS in 02/2014 at an age of 25 years. In 05/2014 she started on dimethylfumarate treatment (Figure 6A). Due to side effects, treatment was changed to glatiramer acetate in 10/2015. In 03/2017 she suffered from a severe relapse and cerebral MRI scan demonstrated new T2 lesions. JCV index was negative and first NAT infusion was performed in 06/2017. After 4 months, cerebral MRI scan presented two new T2 lesions. NAT infusions were continued. In 01/2018 after the 8. NAT infusion, the patient suffered again of an acute relapse that was treated with corticosteroids. Testing and re-testing of antibodies against NAT (nAbs) was done and high titers of nAbs were confirmed in 02/2018 and 03/2018 (testing was performed at the Department of Neurology, University Hospital Bochum, Prof. Dr. R. Gold). Evaluation of JCV index in 11/2017 and 04/2018 presented again negative results. MRI scan presented progress of >10 new T2 lesions in 05/2018. NAT treatment was stopped and treatment with alemtuzumab was started in 06/2018 (Figure 6).

Evaluation of blood cell count demonstrated that in contrast to our cohort presented above leukocytes and lymphocytes were not relevantly changed after starting NAT treatment in this patient (Figures 6B,C). Lymphocytes and its subsets were not increased during the NAT treatment and did not change in the post-treatment period as well (Figures 6E,F). We assume that nAbs developed quite early after NAT start. This case report demonstrates that real world lab data can potentially identify patients with nAbs development to NAT.

### Standard Serological Parameters During NAT Treatment

**Figure 7:**
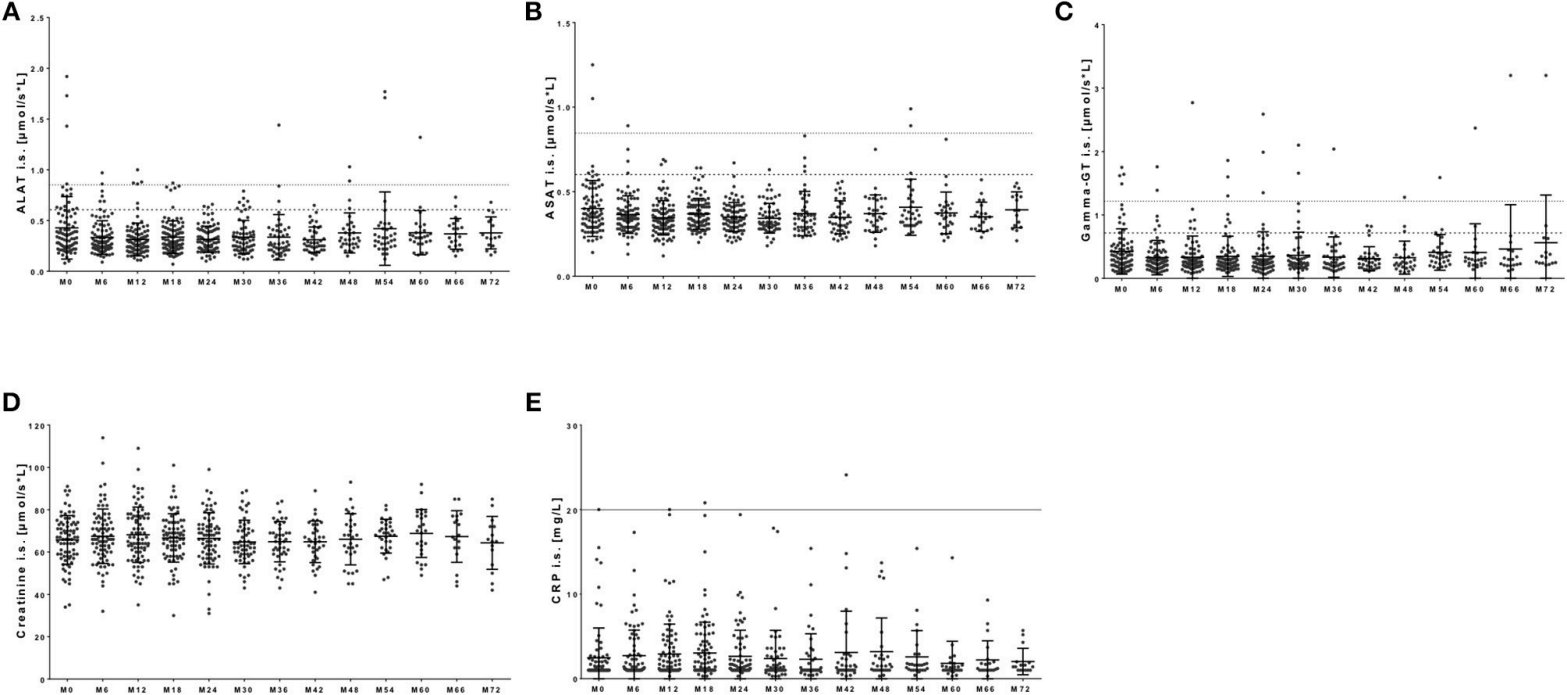
Serological parameters during NAT treatment. Before each infusion started, blood samples were taken and analyzed in a certified medical laboratory. Analysis of serological parameters including ALAT **(A)**, ASAT **(B)**, gamma-GT **(C)**, creatinine **(D)**, and CRP **(E)** were included. Data are shown for baseline analysis (M0) and sixth months follow up (M6, M12, […], M72). Data are presented as mean ± standard deviation. Dotted line indicates the reverence range for men for ALAT and ASAT at a value of 0.85 μmol/s*L and for gamma-GT at 1.2 μmol/s*L. Broken line indicates reverence range for women for ALAT and ASAT at a value of 0.6 μmol/s*L and for gamma-GT at 0.7 μmol/s*L. There were no statistical significant changes.

Standard serological parameters were measured every 3 months as well (Figure 7). In most of the patients, liver enzymes including alanine aminotransferase (ALAT), aspartate aminotransferase (ASAT), and gamma-Glutamyltransferase (gamma-GT) demonstrated levels in the reference range over the whole observation period (Figures 7A–C). Nevertheless, out of our 120 patients few patients presented with transient and asymptomatic increase of ALAT, ASAT or gamma-GT (Figures 7A–C). After NAT initiation, ALAT levels were increased once during the whole observation period but reached reference range at re-test in 16 patients. Seven patients presented with repeated increase of ALAT level. In nine patients increased ASAT levels were seen once during NAT treatment with normalized values at re-testing. One patient presented repeated increase of ASAT levels threw observation. None of these patients reached levels >3x upper normal limit for ASAT, and only one patient reached levels >3x upper normal limit for ALAT, but presented with normalized values after re-test. (Figures 7A,B). Gamma-GT was elevated in 21 patients during NAT therapy. Elevation of gamma-GT was transient and appeared only once in eight of the investigated patients. In 13 patients, repeated elevations of gamma-GT levels were seen. Only three patients presented with gamma-GT levels >3x upper normal limit. This increase was asymptomatic and without any clinical significance.

Kidney function parameters including creatinine (Figure 7D) as well as sodium and potassium levels (data not shown) remained stable within physiological ranges in most of the patients. Only few patients appeared with transient elevation in creatinine levels at least once during NAT therapy in our cohort (Figure 7D). Creatinine levels lower than physiological reference ranged appeared primarily in immobilized patients and did not reflect any clinical relevance (Figure 7D). CRP levels ≥20 mg/dL were defined as clinical significant. In our observation, almost all patients presented with normal CRP levels during the whole observation period (Figure 7E). Nevertheless, 10 patients presented with an increase in CRP up to 144 mg/dL during NAT therapy. Six of these 10 patients showed clinical symptoms of an infection. At onset of CRP increase, six of these 10 patients suffered from respiratory tract infection that resolved during follow up. In four of these patients, CRP increase was paralleled by clinical significant increase of leukocyte but not lymphocyte count.

## Discussion

In our longitudinal observational study, we systematically collected real world lab data including specific immune cell subsets and routine laboratory parameters in NAT treated patients during long-term NAT treatment. Upon NAT treated patients, there are a huge amount of reports available discussing long period outcome and clinical parameters including relapse rate, MRI-activity, and disease progression presenting long-term efficacy (28–30), whereas analyses of immune cell subsets and standard lab analyses are restricted only up to 24–48 months follow up (15, 23, 24). Compared to previous studies, we analyzed a representative large number of patients for a treatment period up to 6 years. Our cohort demonstrated the excellent clinical effectiveness of NAT in the real world scenario as well. NAT selectively directs the α4 subunit of VLA-4 on the surface of leukocytes (2). The interplay of VLA-4 on white blood cells and VCAM-1 expressed on endothelial cells enables peripheral immune cells to cross the blood-brain barrier and boosting CNS inflammation as known in MS pathology (1, 31). While NAT treatment, leukocyte extravasation especially into the CNS is inhibited and immune cells are sequestrated in peripheral blood (13, 21). In line with previous data, our results confirm early increase of peripheral lymphocyte subsets in NAT treated patients (21, 22, 24, 32, 33). Additionally we demonstrate an early and persistent biological response of NAT treatment in different leukocyte subsets over the entire period of 6 years evaluation. Interestingly, pretreatment conditions could not predict the course of leukocytes and lymphocytes after NAT start in our cohort, baseline leukocyte and lymphocyte count was correlated with relative but not absolute increase. Regarding lymphocyte subsets, increase of CD8+ and CD19+ lymphocytes was most pronounced and especially CD19+ B cells were higher than normal level in most of the treated patients. These data were also reported by others (24, 33). Previous observations confirmed the relevance of VLA-4 also on B cells for the CNS recruitment and inflammation in MS pathogenesis (34). Though selective inhibition of VLA-4 dependent B cells may contributes to the efficacy during NAT therapy (34). The risk of nAbs development during NAT therapy is a well-known phenomenon and may interfere with NAT efficacy (35). Here we demonstrate that the typical immunological effects in the periphery are missing in a patient with nAbs to NAT. Though, even standard blood count can probably assist to identify insufficient impact of NAT treatment and to consider further testing. Of course, this has to be systematically proven in a larger cohort.

Previous studies presented various expression levels of VLA-4 on different lymphocyte subtypes with higher levels on B lymphocytes than on T lymphocytes and also more pronounced on CD8+ T cells than on CD4+ T cells (21, 32, 36). Though, the VLA-4/VCAM-1 mediated transmigration of immune cells is differently affected among different peripheral immune cells in NAT treated patients that lead to different sequestration in peripheral blood. Additional to peripheral lymphocyte sequestration due to impaired transmigration mechanisms, effects on the lymphocyte release from bone marrow are discussed (11, 12, 14, 16). Different studies presented mobilization of hematopoietic lymphoid precursor cells from the bone marrow by NAT mediated blocking of retention signals (11, 12, 14). In the line with this, an increase in erythroblasts was seen as well in our cohort. This mobilization additionally contributes to increase of circulating B cells especially with naïve and memory phenotype in NAT treated patients (16). Furthermore, NAT-associated but not clinically relevant morphologic changes in lymphocytes have been described defined by enhanced fraction of atypical lymphocytes (37, 38).

During our observation, CD4/CD8 ratio did not change during the entire period of therapy compared to baseline. These findings are comparable with other results of shorter evaluation periods (24), whereas some early reports presented a decrease in CD4/CD8 ratio suggesting increased risk of opportunistic infections including PML in such patients (23, 39). These studies were usually characterized by smaller samples size and shorter period of evaluation compared to our analysis. In our cohort, NAT lead to increase and re-distribution of peripheral immune cell subsets, which happened early and remained constant without relevant variation during the whole period of 6 years evaluation. Although some immune cell subsets increased out of reference range, no serious adverse events including severe infections or opportunistic infections appeared, no malignancies, or hematological abnormalities were detectable.

Additionally to T and B cell subsets, NK cells increased early directly after NAT initiation. In comparison with other lymphocyte subtypes, NK cells presented one of the most pronounced increases in periphery. These data are in line with other observations that additionally discussed a link between increase of NK cells and response during NAT treatment (21, 24, 40, 41). Here we show, that NAT induced changes in NK cell count persist even years after treatment initiation possible additionally contributing to efficacy in long-term treatment.

Monocytes, eosinophils, and basophils showed a lower but significant increase. In contrast to another study in which monocytes steadily increased over the whole observational period (15), in our analysis monocyte cell count increased to a constant level already after 1 month of treatment start and kept stable. Blocking VLA-4 by NAT leads to sequestration of monocytes and granulocyte subsets in peripheral blood, but does not affect migration of myeloid progenitor cells as seen for the lymphoid progenitors described above (42). These aspects may explain the moderate increase in monocytes and granulocyte subsets compared to lymphocytes. No significant changes were seen in cell count and distribution of neutrophils. Neutrophils do not express VLA-4, therefore, NAT does not affect its distribution (15, 43). Furthermore, we confirmed stable hematological parameters including erythrocyte count, hemoglobin, hematocrit, and platelet count during longterm evaluation of NAT treatment (15). In our evaluation, no association between the distribution of peripheral immune cell subsets and clinical disease activity parameters during NAT treatment could be found. The immune cell phenotyping presented in our study is part of the routine lab testing in our treated MS patients and is characterized by limitations in comparison to more detailed immune cell profiling as presented in other immune profiling studies using e.g., high-dimensional cytometry (44). These studies are more complex in immune cell profiling techniques but may elucidate more details in immune cell patterns and clinical response.

Although controlled data regarding peripheral immune cell subsets within first years of NAT treatment are available, reports on standard testing of serological parameters especially in long-term evaluation are missing. Based on the known excellent safety profile of NAT, hepatic, or kidney dysfunction is not common. Nevertheless, frequent testing of routine lab parameters is recommended and part of the monitoring program applied in NAT treated patients (45). During our observation period of 6 years, there were no relevant or long lasting abnormalities in serological testing of liver enzymes or kidney function. In general, patients presented these parameters in physiological ranges with rare, only transient and not clinical significant increases during the whole period of NAT treatment. Evaluation of CRP levels is a helpful tool to define infectious conditions. NAT therapy does not impair variation of CRP levels as seen by other MS treatments e.g., after alemtuzumab initiation (46, 47). In our cohort, only few patients with transient increase of CRP levels were found. In six out of ten patients with increased CRP levels, a clinical relevant infection was apparent. These data are important to demonstrate that evaluation of CRP levels maybe a helpful tool to identify acute infections even in NAT treated patients when changes in peripheral blood cells are a common phenomenon.

Here, we presented real world lab data on NAT demonstrating the consistent and safe impact of NAT on peripheral immune cell subsets and routine lab parameters within real world conditions and everyday clinical practice. We have already shown the value of real world lab data for fingolimod recently (48). Patients included in our investigation have a different clinical profile compared to those included in randomized clinical trials with limiting inclusion criteria allowing the assessment of real world lab data. The broad and unselected patient population is a great advantage of our study, which does represent the common patient in daily clinical practice. Because of the differences in disease duration, previous medication use and various pre-existing conditions, they describe the real world patient population best. Nevertheless, differences in monitoring procedures and data collection in different centers impact the quality and comparability of such real world data (17). Standardized treatment protocols and monitoring tools (e.g., the MSDS3D software approach) can assist to achieve comparable requirements for patient care as well as data collection even at multiple centers and in every-day clinical practice (18). So NAT real world data on pregnancy are available (49). This standardized collection of real world data and observational studies providing longitudinal information on drug profile and different outcomes in real life are essential to improve decision-making and optimize treatment management.

## Availability of the Data and Material

TZ and KA have full access to all the data in the study and takes full responsibility for integrity of the data and the accuracy of the data analysis. Raw data are available on personal demand.

## Author Contributions

KA and TZ study concept and design. MK acquisition of data. MK, KA, and RH analysis and interpretation of data. MK, KA, and TZ drafting of the manuscript. UP and RH critical revision of the manuscript for important intellectual content. RH and MK statistical analysis.

### Conflict of Interest Statement

KA received personal compensation for from Novartis, Biogen Idec, Roche, Sanofi, and Merck for consulting service. TZ received personal compensation from Biogen Idec, Bayer, Novartis, Sanofi and Teva for consulting services. TZ received additional financial support for research activities from Bayer, Biogen Idec, Novartis, Teva, and Sanofi Aventis. UP received speaker fee from Roche. RH received fee from Sanofi. The remaining authors declare that the research was conducted in the absence of any commercial or financial relationships that could be construed as a potential conflict of interest.

## Abbreviations

ALAT: alanine aminotransferase
ASAT: aspartate aminotransferase
CNS: central nervous system
CRP: C-reactive protein
DMT: disease modifying drug
gamma-GT: gamma-glutamyltransferase
EDSS: Expanded Disability Status Scale
i.v.: intravenously
JCV: John Cunningham virus
MRI: magnet resonance imaging
MS: multiples sclerosis
NAT: natalizumab
NEDA: no evidence of disease activity
NK cells: natural killer cells
PML: progressive multifocal leukoencephalopathy
RR: relapsing remitting
VCAM-1: vascular cell adhesion molecule-1
VLA-4: very late antigen 4.
